# One-Pot Green Regioselesctive Synthesis of γ-Lactones from Epoxides and Ketene Silyl Acetals Using 1,3-Dimethylimidazolium Fluoride as a Recoverable Metal-Free Catalyst

**DOI:** 10.3390/molecules22091385

**Published:** 2017-08-28

**Authors:** Mosadegh Keshavarz, Amanollah Zarei Ahmady, Azar Mostoufi, Neda Mohtasham

**Affiliations:** 1 Department of Gas and Petroleum, Yasouj University, Gachsaran 75918-74831, Iran; chem.mosadegh@gmail.com; 2 Marine Pharmaceutical Science Research Center, Ahvaz JundiShapur University of Medical Sciences, Ahvaz 14536-33143, Iran; 3 Department of Medicinal Chemistry, Ahvaz Jundishapur University of Medical Sciences, Ahvaz 14536-33143, Iran; azarmostoufi@yahoo.com; 4 Pediatric Division, Abuzar Children’s Medical Center, Ahvaz Jundishapur University of Medical Sciences, Ahvaz 14536-33143, Iran; mohtashamneda@gmail.com

**Keywords:** γ-lactone, flourous ionic liquid, one-pot synthesis, catalyst, regioselective

## Abstract

In a straightforward and fast protocol, a mixture of 1,3-dimethylimidazolium fluoride ([DMIM]F) and 1-butylimidazolium tetrafluoroborate ([Hbim]BF_4_) efficiently catalyzed the reaction of epoxides with ketene silyl acetals (KSA) to give various γ-lactones under metal-free conditions. Diverse kinds of the desired γ-lactones were directly prepared with high regioselectivities and yields in a simple one-pot procedure using [DMIM]F as Si–O bond activator and [Hbim]BF_4_ as solvent and acidic ionic liquid catalyst. The ionic liquid mixture was recovered and reused three times and no loss in its activity was observed.

## 1. Introduction

**Figure 1:**
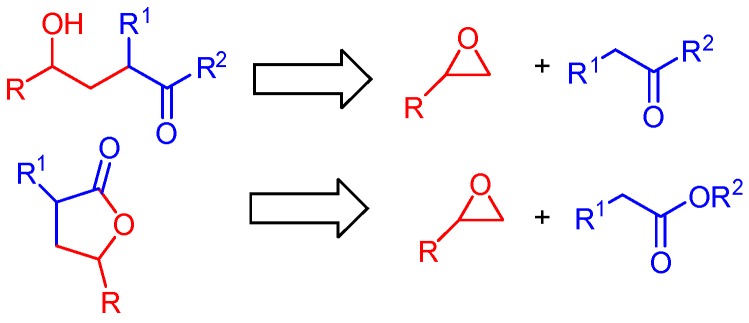
Retro synthesis of homoaldols and γ-lactones.

The reactions of epoxides and carboanion nucleophiles are very important transformations in the synthesis of biologically significant targets [1,2,3]. One of the most important of these reactions is the reaction of enolates of ketones and esters with epoxides [4,5]. However, these enolates are generally unreactive towards epoxides, which generally precludes the application of what would probably be the most efficient approach to homoaldols and γ-lactones (Figure 1).

Transmetallation of lithium ester enolates with diethylaluminum chloride affords the more reactive aluminium enolates, whose reactions with epoxides afford the desired γ-hydroxyesters in moderate to good yields [4,5,6]. In order to avoid the abovementioned problems, indirect methods have been developed, based on the application of diethylethoxyalkynylalane, [4] or silylynamine [7], as the acetate enolate equivalents. Nevertheless, these replacement approaches are not completely efficient because they are limited to the synthesis of α-unsubstituted butanolides, although the latter offers some possibilities to achieve molecular diversity.

**Figure 2:**
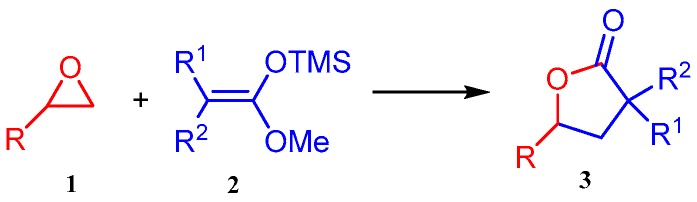
The best choice approach for the preparation of γ-lactones.

The best choice approach for the preparation of γ-lactones which are generally important class of extensive biologically active compounds is the ring opening of epoxides 1 with KSA 2 (Figure 2) [8,9,10].

The use of KSA as an alternative for unreactive ester enolates affords a carboanion reactive toward epoxides to furnish γ-lactone products if an effective catalyst is used. In this regard, to date, few reports on this process exist and in most cases 1.5–3.0 equivalents of Lewis acids such as BF_3_·OEt_2_ [11], TiCl_4_ [12,13], and LiClO_4_ [14] were used as promoters. The main limitations of these methods are related to the use of over-stoichiometric quantities of nucleophiles and Lewis acids. Moreover, working at low temperatures is needed. Furthermore, in the most reported cases, the substrate scope is limited to a specific class of epoxides (epihalohydrins, 1,1-dibromo-3,4-epoxy-1-alkenes) and the reactions give the products in two steps.

The catalytic role of fluoride anion for Si–O bond activation was proved by Vaccaro and co-workers in the reactions that involved the nucleophilic addition of KSA to aldehydes (Mukaiyama aldol reaction) [15,16] and to β-nitrostyrenes (Michael-type addition) to synthesize β-hydroxyesters and γ-nitroesters, respectively, by using Amberlite fluoride resin as fluoride ion source [17]. It was also shown that tetrabutylammonioum fluoride (TBAF) is an effective catalyst for Si-N bond activation when TMSN_3_ reacts with organic nitriles to furnish tetrazole targets [18]. This catalyst was recently reported as the metal-free catalyst and Si–O bond activator for the preparation of γ-lactones from the reaction of KSA and various epoxides under solvent-free conditions [19]. TBAF effectively catalyzed the preparation of γ-lactones in a one-pot procedure. However the catalyst was not recoverable and reusable.

The efficient and high-throughput synthesis of complex organic molecules with lower impact on the environment is one of the most important objectives in modern synthetic organic and green chemistry [20]. Multicomponent reactions (MCRs) combined with the application of task-specific ionic liquids (TSIL) are valuable tools for the environmentally friendly preparation of structurally diverse chemical libraries of drug-like heterocyclic compounds [21,22,23,24].

In this context, we wish to report task-specific ionic liquid, 1,3-dimethylimidazolium fluoride ([Dmim]F) as a new efficient and reusable Si–O bond activator and butylimidazolium tetrafluoroborate ([Hbim]BF_4_) as solvent and acidic ionic liquid catalyst for the preparation of various γ-lactones from the reaction of epoxides with KSA under metal-free conditions. By using the [Dmim]F as fluoride ion-containing ionic liquid, various kinds of γ-lactones were obtained in a fast one-pot and straightforward protocol.

## 2. Results and Discussion

The user friendly and adjustable properties of ionic liquids have prompted numerous applications, not only as environmentally benign reaction media, but also as catalysts [25], task-specific reagents [26] and chirality transfer media [27]. From this perspective, combining the synthetic potential of multi-component reactions (MCRs) with the dual properties of ionic liquids as solvents and promoters has resulted in the development of new and promising eco-compatible organic transformations [21].

**Figure 3:**
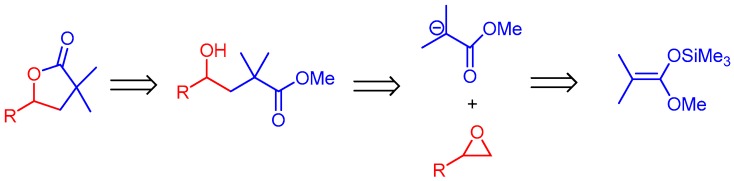
Retrosynthesis strategy for the synthesis of γ-butyrolactones.

In this regard, herein we have investigated the catalytic properties of 1,3-dimethylimidazolium fluoride as a fluoride ion-containing catalyst for Si–O bond activation in the reactions of KSA with epoxides to furnish desired γ-lactones. The retrosynthesis strategy for the preparation of γ-lactones by this procedure is depicted in Figure 3. To approach this goal, we have selected the synthesis of 3,3-dimethyl-5-phenoxymethyl-dihydro-furan-2-one (**3a**) via addition of phenyl glycidyl ether (**1a**) with KSA (**2**) as model reaction (Table 1).

In an initial attempt, it was seen that in the absence of the [Dmim]F the reaction did not proceed, even after 72 h (Table 1, entry 1). Using *n*-butylimidazolium tetrafluoroborate ([Hbim]BF_4_) in the absence of [Dmim]F also did not furnish any product after 72 h (Table 1, entry 2). By using equivalent molar ratios of **1a** and **2** (1:1) in the presence of 2% mol of [Dmim]F under solvent free conditions at 60 °C, the desired γ-butyrolactone **3a** was obtained in 15% isolated yield after 5 h (Table 1, entry 3). Increasing the molar ratio of **2** to 1.5 mmol and the molar percent of the [Dmim]F to 5%, the product was obtained after 2 h in 90% isolated yield (Table 1, entry 8).

**Figure 4:**
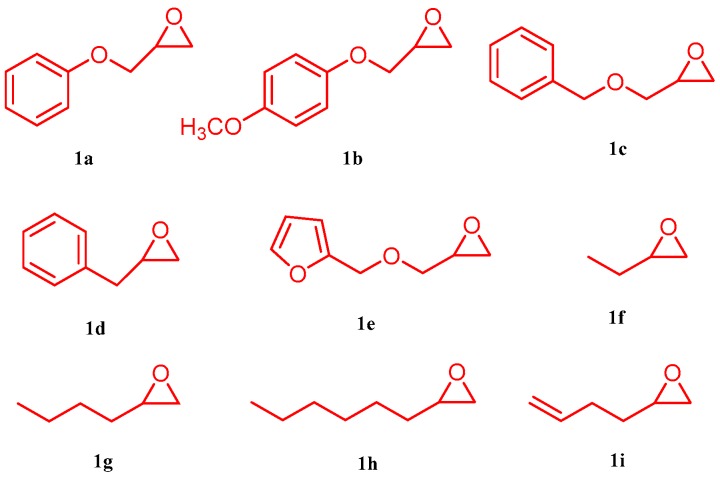
Selected epoxides for the reaction with KSA.

The best result was obtained when 5 mol % of [Dmim]F was used as catalyst in [Hbim]BF_4_ as solvent. The yield was enhanced to 98% and the reaction time decreased to 0.25 h (Table 1, entry 9). Greater amount of the catalyst and higher reaction temperatures did not significantly improve the yield and reaction rate (Table 1, entry 9 vs. 10, 11). The optimized reaction clearly indicates the role of [Dmim]F as catalyst and [Hbim]BF_4_ as reaction medium to achieve the best conditions. To extend the scope of the reaction and to generalize the procedure, various structurally diverse epoxides shown in Figure 4 were reacted with KSA (2) under the optimized reaction conditions.

**Figure 5:**
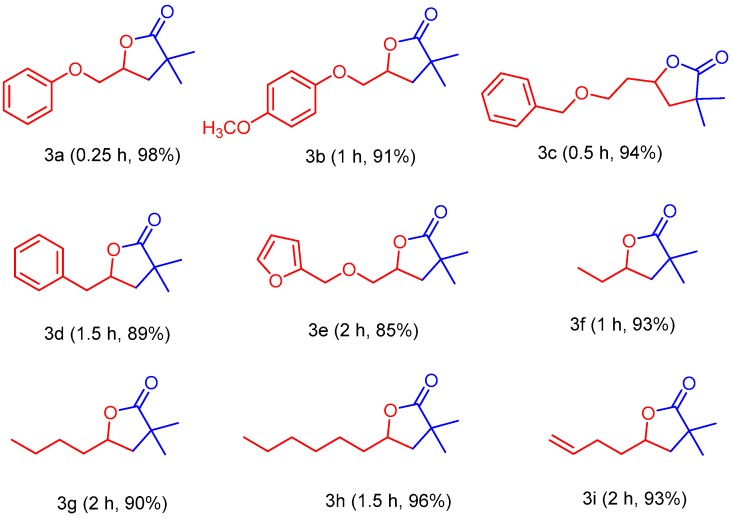
Prepared γ-lactones from the reaction of epoxides with KSA using task-specific [Dmim]F/[Hbim]BF_4_ ionic liquid mixture.

The structures of all prepared γ-lactones compounds were ascertained by their satisfactory elemental analyses and spectral (IR, ^1^H-, ^13^C-NMR, and MS) studies. In the all cases, in the presence of [Dmim]F and [Hbim]BF_4_, the ring opening of the epoxides **1a**–**1i** and then, direct in situ cyclization were consecutively observed which finally led to the formation of desired γ-lactones (Figure 5 (**3a**–**3i**)).

**Scheme 1:**
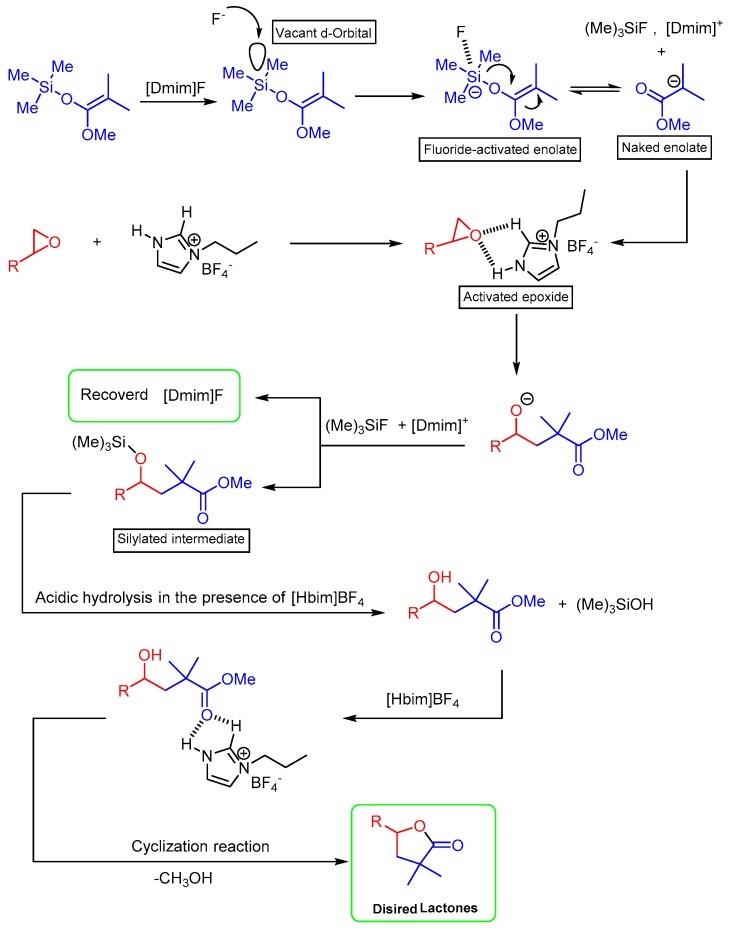
Proposed mechanism for the preparation of γ-lactones using task specific [Dmim]F/[Hbim]BF_4_ ionic liquid mixture.

The proposed mechanism for the synthesis of γ-lactone compounds **3** in the presence of [Dmim]F and [Hbim]BF_4_ is shown in Scheme 1. We envisioned that this reaction could be realized in a one-pot, two-step manner. Initially, [Dmim]F as fluoride-containing catalyst injects its electrons to the vacant d-orbital of KSA to furnish a fluoride-activated enolate which is in equilibrium with the naked enolate. In another catalytic reaction, [Hbim]BF_4_ as an acidic ionic liquid, activates the epoxide by the formation of hydrogen-bonding using its two imidazolum acidic hydrogens with the epoxide [28,29]. Then, the naked enolate performs a nucleophilic attack on β-carbon of the activated epoxide followed by the reaction with trimethylsilyl fluoride (formed from the first reaction) that prepares the silylated intermediate and also recovers the [Dmim]F catalyst. According to the reported literature [15], Si and O, particularly when O is in anionic form, are very attracted toward each other. On the other hand, when the enolate reacts with trimethylsilyl fluoride, [Dmim]^+^ is ready as a powerful cationic source to capture the leaving F^−^, to regenerate the [Dmim]F catalyst. The silylated intermediate, in the presence of [Hbim]BF_4_ underwent acidic hydrolysis leading to a methyl β-hydroxy ester which in the next step became activated by [Hbim]BF_4_ to furnish the desired γ-butyrolactone in a direct cyclization step. This proposed mechanism accounts for the role of [Dmim]F as a Si–O bond activator in the first step and the catalytic activity of [Hbim]BF_4_ for activation of epoxide and the lactonization step.

The ring opening and subsequent direct in situ cyclization which were accomplished by this procedure are some of the superior features of the introduced method to previously mentioned procedures [4,5] in which, after Lewis acid-promoted ring opening of the epoxide, addition of *p*-TsOH to the reaction mixture is needed to obtain the cyclized product **3**.

In addition, in organic synthesis, employing an excess amount of reagents is not cost effective and additional complicated purification is needed. Herein, other than the straightforward experimental protocol provided by [Dmim]F/[Hbim]BF_4_, the reaction does not require the use of wasteful over-stoichiometric amounts of promoters and additional additives. This procedure combined green conditions and (metal-free) fluoride catalysis to produce an efficient and superior method for the preparation of various γ-butyrolactones with complete β-regioselectivity. In all the cases, with both aromatic and aliphatic epoxides, only the corresponding lactone products were isolated in high to excellent yields (85–98%) at short reaction times (0.25–2 h).

The recovery of a catalyst is highly preferable for a greener process. For this purpose, the reusability of [Dmim]F/[Hbim]BF_4_ mixture was studied for third consecutive cycles (fresh + two cycles) for the synthesis of 3,3-dimethyl-5-phenoxymethyl-dihydro-furan-2-one (**3a**). From Table 2, entries 1–3, it can be seen that [Dmim]F/[Hbim]BF_4_ can be reused up to at least three runs without need to reload and the yield difference between the first and 3th runs is only 6%, which indicated that the catalyst efficiency is almost completely maintained during three consecutive runs. In addition, using 1,3-dimethylimidazolium chloride, bromide or iodide instead of 1,3-dimethyl imidazolium fluoride catalyst did not lead to any product even after 72 h (Table 2, entries 4–6). These observations clearly indicate the critical and powerful role of fluoride anion as a Si–O bond activator [15,16,17,18,19].

## 3. Materials and Methods

### 3.1. General Information

NMR spectra were acquired on a Bruker Avance III NMR spectrometer (Bruker AMX-400 NMR spectrometer) with the magnetic field of 11.74 Tesla. HR-ESI-MS were acquired on a Bruker Q-TOF mass spectrometer. The IR spectra of the compounds were recorded using an IRAffinity-1S Fourier transform IR (FTIR) spectrometer (Shimadzu, Tokyo, Japan). Chemical purities of the compounds were checked by classical TLC applications performed on silica gel 60 F254 (Merck KGaA, Darmstadt, Germany). The deuterated chloroform (CDCl_3_, deuterated ratio, 99.8%) with TMS as the internal referent were produced by Cambridge Isotope Labo-ratories, Inc. (Andover, MA, USA). All other chemicals were obtained either from Sigma-Aldrich (Sigma-Aldrich Corp., St. Louis, MO, USA) or Merck (Merck KGaA, Darmstadt, Germany) and used without further purification. 

### 3.2. Preparation of 1,3-Dimethylimidazolium Fluoride ([Dmim]F)

A solution of AgF (5 mmol) dissolved in H_2_O (50 mL) was added slowly to a stirred solution of 1,3-dimethylimidazolium iodides (5 mmol) in H_2_O (50 mL). After stirring at room temperature for 3 h, the mixture was filtrated and the water was evaporated under reduced pressure at room temperature. The crude residue was dissolved in anhydrous methanol. The subsequent filtration and the evaporation of the solvent under reduced pressure gave [Dmim]F in 76% yield [20].

### 3.3. General Procedure for the Synthesis of γ-Lactones Using 1,3Dimethylimidazolium Fluoride as Catalyst 

[Dmim]F (0.006 g, 0.05 mmol), epoxide (2 mmol) and 1-methoxy-2-methyl-1-(trimethylsiloxy)-propene (0.523 g, 3 mmol) were added in a screw capped vial equipped with [Hbim]BF_4_ (1 mL) and a magnetic stirrer. The resulting mixture was left under vigorous stirring at 60 °C. After completion of the reaction as indicated by Thin Layer Chromatography (petroleum ether:ethyl acetate 6:1), the reaction mixture was cooled to room temperature then the organic phase was extracted with diethyl ether (10 mL) and loaded on a silica gel column chromatography (petroleum ether:ethyl acetate 6:1) to isolate the pure desired γ-lactone. The recovered catalyst was stored for another consecutive reaction run.

### 3.4. Selected Spectral Data 

*3,3-Dimethyl-5-phenoxymethyl-dihydrofuran-2-one* (**3a**). IR (neat): ν = 1781(CO) cm^−1^. ^1^H-NMR (400 MHz, CDCl_3_): δ = 7.31–7.26 (2H, m), 6.98 (2H, t, *J* = 7.3 Hz), 6.91 (2H, d, *J* = 8.0 Hz), 4.80–4.78 (1H, m), 4.1620134.08 (2H, m), 2.23 (1H, dd, *J*_1_ = 12.8, *J*_2_ = 6.7 Hz), 2.12–2.06 (1H, m), 1.35 (3H, s), 1.32 (3H, s). ^13^C-NMR (100 MHz, CDCl_3_): δ = 181.4, 158.2, 129.6, 121.4, 114.6, 74.4, 69.0, 40.0, 39.2, 25.1, 25.0. GC-EIMS (*m*/*z*, %): 220 (M^+^, 47), 113 (35), 94 (45), 85 (100), 77 (46), 57 (40), 55 (31).

*5-(4-Methoxyphenoxymethyl)-3,3-dimethyldihydrofuran-2-one* (**3b**). IR (neat): ν = 1778(CO) cm^−1^. ^1^H-NMR (400 MHz, CDCl_3_): δ = 6.87–6.81 (4H, m), 4.78–4.74 (1H, m), 4.11−4.03 (2H, m), 3.77 (3H, s), 2.21 (1H, dd, *J*_1_ = 12.80 Hz, *J*_2_ = 6.7 Hz), 1.33 (6H, s). ^13^C-NMR (100 MHz, CDCl_3_): δ = 181.4, 154.4, 152.4, 115.8, 114.7, 74.6, 70.0, 55.7, 40.0, 39.2, 25.1, 25.0. GC-EIMS (*m/z*, %): 250 (M^+^, 43), 124 (66), 123 (20), 109 (22), 81 (19), 43 (100).

*5-Benzyloxymethyl-3,3-dimethyldihydrofuran-2-one* (**3c**). IR (neat): ν = 1770(CO) cm^−1^. ^1^H-NMR (400 MHz, CDCl_3_): 7.45–7.25 (5H, m), 4.65–4.53 (3H, m), 3.66 (1H, dd, *J*_1_ = 10.9 Hz, J_2_ = 3.5 Hz), 3.59 (1H, dd, *J*_1_ = 10.9 Hz, *J*_2_ = 5.4 Hz), 2.09 (1H, dd, *J*_1_ = 12.7 Hz, *J*_2_ = 6.6 Hz), 2–1.90 (1H, m), 1.28 (3H, s), 1.27 (3H, s) ^13^C-NMR(100 MHz, CDCl_3_): δ = 187.7, 137.7, 128.5, 127.8, 127.7, 75.7, 73.6, 71.4, 40.0, 39.2, 25.0, 24.9.

*5-Benzyl-3,3-dimethyldihydrofuran-2-one* (**3d**). IR (neat): ν = 1775(CO) cm^−1^. ^1^H-NMR (400 MHz, CDCl_3_): δ = 7.40–7.18 (5H, m), 4.70–4.58 (1H, m), 3.10 (1 dd, *J*_1_ = 13.9, J_2_ = 6.5 Hz), 2.87 (1H, dd, *J*_1_ = 13.9, *J*_2_ = 6.2), 2.08 (1H, dd, *J*_1_ = 12.7, *J*_2_ = 5.9 Hz), 1.88–1.77 (1H, m), 1.23 (3H, s), 1.22 (3H, s). ^13^C-NMR (100 MHz, CDCl_3_): δ = 183.6, 141.5, 129.4, 128.6, 127.0, 41.6, 43.0, 41.6, 38.2, 25.0, 24.4. GC-EIMS (*m/z*, %): 204 (M^+^, 14), 113 (66), 91 (62), 85 (100), 57 (49).

*5-(Furan-2-ylmethoxymethyl)-3,3-dimethyldihydrofuran-2-one* (**3e**). IR (neat): ν = 1772(CO) cm^−1^. ^1^H-NMR (400 MHz, CDCl_3_): δ = 7.44 (1H, s), 7.37–7.36 (2H, m), 4.62–4.58 (1H, m), 4.56 (2H, s), 3.76 (1H, dd, *J*_1_= 10.8, *J*_2_ = 3.2 Hz ), 3.66 (1H, dd, *J*_1_ = 10.8 Hz, *J*_2_ = 5.2 Hz), 2.10 (1H, dd, *J*_1_ = 6.4 Hz, *J*_2_ = 12.80 Hz), 1.96–1.91 (1H, m), 1.29 (6H, s). ^13^C-NMR (100 MHz, CDCl_3_): δ = 181.7, 151.2, 143.0, 110.3, 109.8, 75.6, 71.1, 65.3, 40.0, 39.2, 25.0, 24.8.

*5-Ethyl-3,3-dimethyldihydrofuran-2-one* (**3f**). IR (neat): ν = 1777 (CO) cm^−1^. ^1^H-NMR (400 MHz, CDCl_3_): δ = 4.39–4.35 (1H, m), 2.16 (1H, dd, *J*_1_ = 12.6, *J*_2_ = 5.8 Hz), 1.80–1.62 (3H, m), 1.27 (6H, s), 1.03–1.00 (3H, t, *J* = 7.6 Hz). ^13^C-NMR (100 MHz, CDCl_3_): δ = 182.1, 78.3, 43.1, 40.5, 25.1, 24.5. 9.5. GC-EIMS (*m/z*, %): 113 (34), 85 (54), 69 (100), 56 (71), 43 (15).

*5-Butyl-3,3-dimethyldihydrofuran-2-one* (**3g**). IR (neat): ν = 1771(CO) cm^−1^. ^1^H-NMR (400 MHz, CDCl_3_): δ = 4.45–4.35 (1H, m), 2.14 (1H, dd, *J*_1_ = 12.7, *J*_2_ = 5.9 Hz), 1.80–1.68 (2H, m), 1.62–1.56 (1H, m), 1.52–1.30 (4H, m), 1.27 (3H, s), 1.25 (3H, s), 0.93 (3H, t, *J* = 6.9 Hz). ^13^C-NMR (100 MHz, CDCl_3_): δ = 181.7, 77.2, 75.6, 65.3, 39.2, 29.7, 25.0, 24.8, 15.0. GC-EIMS (*m/z*, %): 113 (57), 85 (72), 69 (100), 56 (91), 43 (17).

*5-Hexyl-3,3-dimethyldihydrofuran-2-one* (**3h**). IR (neat): ν = 1780(CO) cm^−1^. ^1^H-NMR (400 MHz, CDCl_3_): δ = 4.50–4.35 (1H, m), 2.16 (1H, dd, *J*_1_ = 13.0, *J*_2_ = 5.5 Hz), 1.80–1.66 (2H, m), 1.65–1.53 (1H, m), 1.52–1.40 (1H, m), 1.40–1.20 (13H, m), 0.90 (3H, t, *J* = 6.2 Hz). ^13^C-NMR (100 MHz, CDCl_3_): δ = 182.1, 77.2, 43.6, 35.8, 29.7, 29.0, 25.3, 24.5, 22.5, 14.1. GC-EIMS (*m*/*z*, %): 113 (53), 85 (59), 69 (97), 56 (100).

*5-(But-3-enyl)-3,3-dimethyldihydrofuran-2-one* (**3i**). IR (neat): ν = 1775(CO) cm^−1^. ^1^H-NMR (400 MHz, CDCl_3_): δ = 8.80–5.70 (1H, m), 5.10–4.92 (2H, m), 4.50–4.32 (1H, m), 2.34–2.10 (3H, m), 1.92–1.60 (3H, m), 1.27 (3H, s), 1.25 (3H, s). ^13^C-NMR (100 MHz, CDCl_3_): δ = 181.9, 137.1, 115.6, 76.3, 43.5, 40.4, 34.9, 29.6, 25.1, 24.5. GC-EIMS (*m/z*, %): 113 (24), 88 (31), 85 (38), 83 (29), 82(35), 81 (61), 69 (30), 67 (41), 57 (38), 56 (41), 55 (100).

## 4. Conclusions

In conclusion, [Dmim]F/[Hbim]BF_4_ is introduced as task-specific ionic liquid mixture for the preparation of various γ-butyrolactones with complete β-regioselectivity. [Dmim]F/[Hbim]BF_4_ provided a straightforward experimental protocol for the preparation of desired γ-lactones and the reactions do not require the use of wasteful over-stoichiometric amounts of promoters and additional additives. This procedure combines green and metal-free conditions to achieve an efficient and superior method for the preparation of various γ-butyrolactones. The task specific ionic liquid mixture can be reused up to at least three runs without need to reload and the products were isolated in high to excellent yields.

## Figures and Tables

**Table 1:**
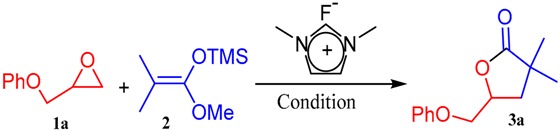
Investigation of catalytic activity of [Dmim]F for the synthesis of γ-butyrolactones under various conditions.

Entry	Catalyst (mol %)	Solvent	1a:2 (mmol:mmol)	T (°C)	Time (h)	Yield (%) ^a^
1	-	-	1:1	60	72	0
2	-	[Hbim]BF_4_	1:1.5	60	72	0
3	[Dmim]F(2%)	-	1:1	60	5	15
4	[Dmim]F(2%)	-	1:1.5	60	5	30
5	[Dmim]F(2%)	-	1.5:1	60	5	20
6	[Dmim]F(5%)	-	1:1	60	5	40
7	[Dmim]F(5%)	-	1.5:1	60	5	45
8	[Dmim]F(5%)	-	1:1.5	60	2	90
9	[Dmim]F(5%)	[Hbim]BF_4_	1:1.5	60	0.25	96
10	[Dmim]F(5%)	[Hbim]BF_4_	1:1.5	80	0.25	98
11	[Dmim]F(10%)	[Hbim]BF_4_	1:1.5	60	0.25	97

^a^ isolated yields.

**Table 2:**
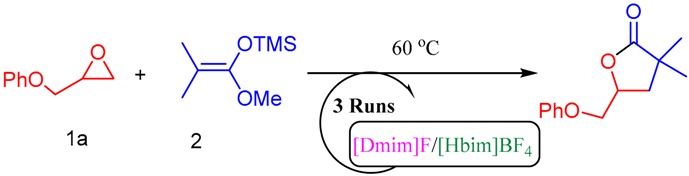
Recycling study of ionic liquids mixture and efficiency of 1,3-dimethylimidazolium fluoride in comparison with other 1,3-dimethylimidazolium halides ^a^.

Entry	Catalyst/Solvent System	Run	Tim (h)	Yield(%) ^b^
1	[Dmim]F (5%)/[Hbim]BF_4_	1	0.25	96
2	Recovered from 1st Run	2	0.25	95
3	Recovered from 2nd Run	3	0.25	90
4	Recovered from 3rd Run	4	0.25	73
5	[Dmim]Cl (5%)/[Hbim]BF_4_	1	72	0
6	[Dmim]Br (5%)/[Hbim]BF_4_	1	72	0
7	[Dmim]I (5%)/[Hbim]BF_4_	1	72	0

^a^ Reaction conditions: 1a:2 (1:1.5), 60 °C. ^b^ Isolated Yields.
